# Identification of adults with sepsis in the prehospital environment: a systematic review

**DOI:** 10.1136/bmjopen-2016-011218

**Published:** 2016-08-05

**Authors:** Michael A Smyth, Samantha J Brace-McDonnell, Gavin D Perkins

**Affiliations:** 1 Clinical Trials Unit, University of Warwick, Coventry, UK; 2 Midlands Air Ambulance, Stourbridge, UK; 3 West Midlands Ambulance Service NHS Foundation Trust, Dudley, UK; 4 Heart of England Hospital NHS Foundation Trust, Birmingham, UK

**Keywords:** sepsis, screening tool, prehospital, EMS, paramedic

## Abstract

**Objective:**

Early identification of sepsis could enable prompt delivery of key interventions such as fluid resuscitation and antibiotic administration which, in turn, may lead to improved patient outcomes. Limited data indicate that recognition of sepsis by paramedics is often poor. We systematically reviewed the literature on prehospital sepsis screening tools to determine whether they improved sepsis recognition.

**Design:**

Systematic review. The electronic databases MEDLINE, EMBASE, CINAHL, the Cochrane Library and PubMed were systematically searched up to June 2015. In addition, subject experts were contacted.

**Setting:**

Prehospital/emergency medical services (EMS).

**Study selection:**

All studies addressing identification of sepsis (including severe sepsis and septic shock) among adult patients managed by EMS.

**Outcome measures:**

Recognition of sepsis by EMS clinicians.

**Results:**

Owing to considerable variation in the methodological approach adopted and outcome measures reported, a narrative approach to data synthesis was adopted. Three studies addressed development of prehospital sepsis screening tools. Six studies addressed paramedic diagnosis of sepsis with or without use of a prehospital sepsis screening tool.

**Conclusions:**

Recognition of sepsis by ambulance clinicians is poor. The use of screening tools, based on the Surviving Sepsis Campaign diagnostic criteria, improves prehospital sepsis recognition. Screening tools derived from EMS data have been developed, but they have not yet been validated in clinical practice. There is a need to undertake validation studies to determine whether prehospital sepsis screening tools confer any clinical benefit.

**Strengths and limitations of this study**

- Despite using very broad search criteria, little robust evidence regarding prehospital sepsis screening was identified.
- The studies found employed disparate methodologies, exhibit significant heterogeneity, generally involve small numbers of patients (limiting the precision of reported results) and were invariably of very low quality.
- The conclusions that can be drawn from this systematic review are, therefore, limited and findings should be interpreted with caution.

## Introduction

Sepsis is a systemic response to infection, which may progress to severe sepsis and septic shock.^1^ In the UK, there are an estimated 102 000 cases of severe sepsis each year resulting in >37 000 deaths.^2^ It has been reported that more than two-thirds of severe sepsis cases are initially seen in the emergency department (ED)^3^ and around half of ED sepsis patients arrive by ambulance.^4–9^ Patients with sepsis arriving at the ED via emergency medical services (EMS) are likely to be sicker than those arriving by other means,^5^ ^7–10^ and up to 80% of patients with severe sepsis admitted to intensive care from the ED will have been transported by EMS.^11^

Although the burden of sepsis upon ambulance services is not well understood, data from Guerra *et al*^12^ suggested that 6.9% of EMS transports were for patients with infection. It is further estimated that 8–10% of EMS patients who have infection will be diagnosed with sepsis.^12^ ^13^ Following a 10-year observational study, Seymour *et al*^14^ reported the incidence of severe sepsis in a North American EMS system to be 3.3 per 100 ambulance transports. Extrapolation of data reported by McClelland and Jones^15^ suggests a lower incidence of sepsis cases in one region of the UK, of ∼1.8% of EMS calls.

In-hospital data indicate that early identification and initiation of treatment of severe sepsis is associated with reduced mortality.^2^ ^16^ It has been argued that there is an opportunity for ambulance clinicians to improve outcomes for this population in the same manner as they do with other time critical, life-threatening conditions such as acute myocardial infarction,^17^ stroke^18^ and major trauma.^19^ Early recognition of sepsis by ambulance clinicians could reduce time to delivery of a limited number of interventions prior to arrival at the ED; however, evidence suggests that recognition of sepsis by paramedics is often poor.^7^ ^12^ ^20–22^ Use of a prehospital sepsis screening tool has been advocated, suggesting that it would lead to improved recognition, and potentially earlier initiation of key interventions such as fluid resuscitation and antibiotic administration prior to arriving at hospital.^5^ ^23^ ^24^

## Objective

The objective of this study was to determine whether, among adult patients presenting to EMS, the use of a prehospital sepsis screening tool by ambulance clinicians, compared to ambulance clinician judgement alone, improves identification of sepsis.

## Design

We followed the Grading of Recommendations, Assessment, Development and Evaluation (GRADE) Working Group methodology^25^ to conduct the review and Preferred Reporting Items for Systematic Reviews and Meta-Analyses recommendations to report our findings.^26^ The review is registered with the International Prospective Register of Systematic Reviews (CRD42014007654).

## Setting

Adult patients managed by EMS in the prehospital environment.

## Study selection

### Electronic searches

We searched MEDLINE, EMBASE, CINAHL, the Cochrane Library and PubMed. No language restrictions were placed. Conference proceedings/meeting abstracts were included to capture grey literature.

### Search terms/search strategy

Search strategies were developed for each database, starting with MEDLINE (see online supplementary appendix 1). The MEDLINE search strategy was adapted for each subsequent database. Initial searches were conducted in July 2014 with a second search completed at the end of June 2015 (including articles published up to 28 June 2015).

10.1136/bmjopen-2016-011218.supp1 supplementary appendix

### Inclusion criteria

- *Language*: no restrictions were placed.
- *Publication type*: original research published in peer-reviewed journals and conference proceedings.
- *Study design*: systematic reviews, meta-analyses, randomised controlled trials, case–control studies, cohort studies and cross-sectional studies.
- *Study population*: adult patients managed by EMS. Populations could comprise a mix of adult and child participants if results were reported separately.
- *Case definition*: no restrictions as to severity of sepsis.

### Exclusion criteria

- *Publication type*: narrative/literature reviews, letters, editorials, commentaries, books and book chapters, lectures and addresses, and consensus statements.
- *Study design*: case reports and qualitative studies.
- *Study population*: In-hospital studies. Mixed adult and child population without distinct reporting, child population and animals.

### Other

Reference lists of included manuscripts were scrutinised. Subject experts were contacted to identify studies missed by electronic searches.

### Data collection and analysis

Studies were screened in two stages. In the first stage, two reviewers (MAS and SJB-M) independently reviewed each citation and abstract against the inclusion criteria. Citations rated as ‘include’ by either reviewer were considered relevant, and citations rated as ‘exclude’ by both reviewers were rejected. In the second stage, the full manuscripts of included citations were again independently screened by two reviewers (MAS and SJB-M) rating each manuscript as ‘include’, ‘maybe’ or ‘exclude’ against the inclusion criteria. If both reviewers rated a manuscript as ‘include’, it was automatically included for critical appraisal. If both reviewers rated a manuscript as ‘exclude’, it was automatically rejected. Where a manuscript was selected as ‘include’ by a single reviewer, or was selected as ‘maybe’ by one or both reviewers, the reviewers discussed if the manuscript should be included or excluded. If the reviewers were unable to agree, a third independent reviewer (GDP) was available to adjudicate.

## Outcome measures

We included any study that reported prehospital sepsis screening or development of prehospital sepsis screening tools and compared accuracy of prehospital diagnosis with in-hospital diagnosis.

## Results

Database searches yielded 4366 citations. Duplicate citations were removed manually within EndNote (V.X7 Thompson Scientific, Carlsbad, California, USA) by a single reviewer (MAS) providing 2958 unique citations. After the first stage of screening, 78 citations were retained and 2880 citations were rejected. Inter-rater agreement for first-stage screening, calculated using Cohen’s κ statistic, was 0.87 (95% CI 0.81 to 0.92). During the second stage of screening, 78 manuscripts were reviewed, 70 were discarded following assessment and 8 were retained for critical appraisal. Inter-rater agreement for second-stage screening, calculated using Cohen’s κ statistic, was 0.82 (95% CI 0.68 to 0.97).

**Figure 1:**
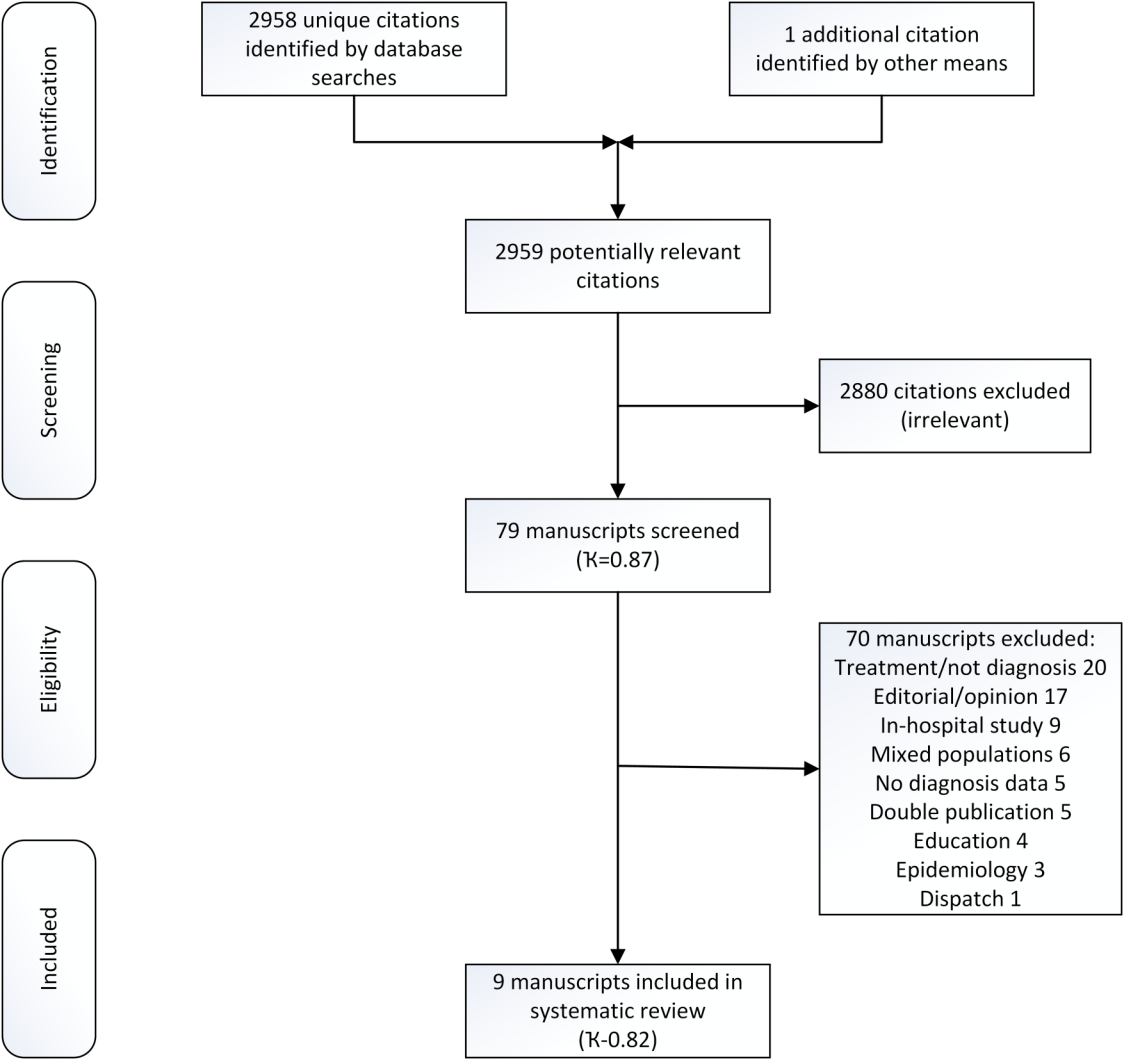
PRISMA flow chart.

No additional citations were identified by scrutinising the reference lists of included manuscripts. One additional study,^15^ a manuscript pending publication, was identified by contacting subject experts. In total, nine studies are included in the final analysis (see figure 1 and online supplementary material).

10.1136/bmjopen-2016-011218.supp2 supplementary material

### Characteristics of included studies

No randomised controlled trials were identified; all included studies were observational in nature. Three studies were published in abstract form only.^20^ ^27^ ^28^ Studies originate from five countries, comprising a total of 147 320 patients. All studies were published in English. The median year of publication was 2013. The data from included studies were extracted and entered into relevant tables by a single reviewer (MAS) and verified by a second reviewer (SJB-M).

Three studies were concerned with derivation of screening tools.^29–31^ Six studies addressed the identification of sepsis within EMS.^12^ ^15^ ^20^ ^21^ ^27^ ^28^ Collectively, six prehospital screening tools were identified in the course of the review (critical illness score,^32^ Prehospital Recognition of Severe Sepsis (PRESS) score,^31^ Prehospital Early Sepsis Detection (PRESEP) score,^30^ Robson tool,^21^ ^30^ modified Robson tool^15^ and BAS 90-30-90^21^ ^30^); a single study reported the accuracy of the Modified Early Warning Score (MEWS).^30^ None of the studies were prospective and no studies were designed specifically to validate a prehospital sepsis screening tool in clinical practice.

All studies used hospital sepsis diagnosis as the reference standard; however, hospital diagnosis was variably determined by Surviving Sepsis Campaign diagnostic criteria, International Classification of Disease coding, ED diagnosis (without description of how diagnosis was determined) or discharge diagnosis (without description of how diagnosis was determined).

### Risk of bias

Bias within observational studies was assessed across the domains of failure to develop and apply appropriate eligibility criteria (inclusion of control population), flawed measurement of exposure and outcome, failure to adequately control confounding and incomplete follow-up. Two reviewers (MAS and SJB-M) independently assessed each article across the bias domains with each being rated as high risk, low risk or level of risk unclear as per GRADE recommendations.^33^ Studies with high risk in one or more domains were considered to be at high risk of bias overall. Similarly studies with low risk for all domains were considered to be at low risk of bias overall. Otherwise, studies were considered to have an unclear risk of bias. Risk of bias assessments are reported in table 1.

**Table 1:** Risk of bias

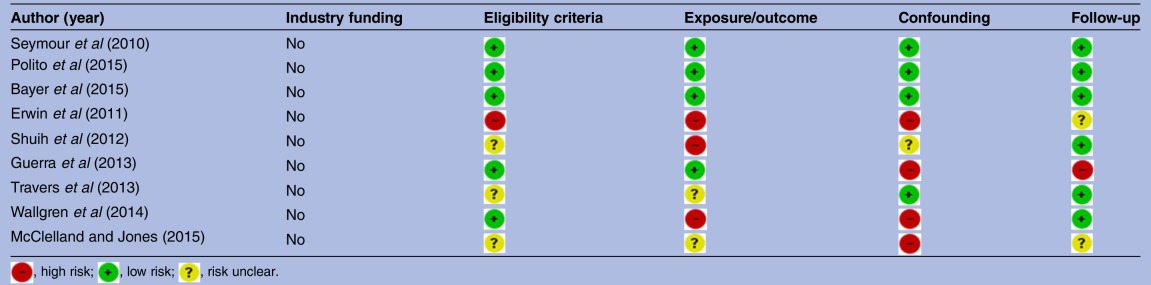

### Quality of evidence

Study design informed initial quality assumptions. No randomised controlled trials were identified. Non-randomised (observational) studies were initially presumed to be ‘low quality’. Two reviewers (MAS and SJB-M) appraised each study across the five core GRADE domains of risk of bias,^33^ inconsistency,^34^ indirectness,^35^ imprecision^36^ and other considerations (including publication bias)^37^ (see online supplementary material). Where concerns were identified, it lowered the overall quality assumptions. Similarly, quality could have been adjusted upward if, for example, a large treatment effect or dose–response had been noted, which subsequently raised our confidence in the estimate of effect.^38^ Quality of evidence, across each outcome of interest, is reported as follows (table 2):

- *High quality*: We are very confident that the true effect lies close to that of the estimate of effect.
- *Moderate quality*: We are moderately confident in the effect estimate: the true effect is likely to be close to the estimate of effect, but there is a possibility that it is substantially different.
- *Low quality*: Our confidence in the effect is limited: the true effect may be substantially different from the estimate of the effect.
- *Very low quality*: We have very little confidence in the effect estimate: the true effect is likely to be substantially different from the estimate of effect.

**Table 2:** Summary of findings

No. of studies	No. of patients	Study design	Risk of bias	Inconsistency	Indirectness	Imprecision	Other	Findings	Level of evidence
*Derivation of prehospital sepsis screening tools*									
3	145, 843	Non-RCT	None	None	Not serious*	Very serious†	None	Seymour *et al*^29^ ^32^ CIS: risk of sepsis 0.76 (95% CI 0.75 to 0.77)	⊕⊙⊙⊙ very low
								Polito *et al* 31 PRESS score: sensitivity 0.85, specificity 0.47, PPV 0.19, NPV 0.96 (95% CI not reported). PRESS score ≥3; sensitivity 0.81; specificity 0.63	
								Bayer *et al*^30^ PRESEP score: sensitivity 0.85 (95% CI 0.77 to 0.92), specificity 0.86 (95% CI 0.82 to 0.90), PPV 0.66, NPV 0.95	
*Sepsis recognition by EMS (using a screening tool)*									
2	161	Non-RCT	Very serious‡	None	Not serious§	Very serious¶	Very serious**	Guerra *et al*^12^ Screening based on SSC criteria identified 32/67 patients with sepsis (47.8%) (95% CI not reported)	⊕⊙⊙⊙ very low
								McClelland and Jones^15^ Screening using modified Robson tool. Sensitivity and specificity for sepsis 43% (95% CI 28% to 58%) and 14% (95% CI 0% to 40%), respectively. Sensitivity and specificity for severe sepsis 30% (95% CI 12% to 47%) and 77% (95% CI 60% to 95%)	
*Retrospective application of EMS data to screening tool by researcher*									
2	728	Non-RCT	Very serious‡	None	Not serious§	Very serious¶	None	Wallgren *et al*^21^ Retrospective application of two different screening tools in comparison to clinical judgement. For sepsis, Robson tool: sensitivity 75% (p<0.001), BAS 90-30-90: sensitivity 43% (p<0.001), clinical judgement: 12% accuracy (95% CI not reported). For severe sepsis, Robson tool: sensitivity 93% (p<0.001), BAS 90-30-90: sensitivity 70% (p<0.001), clinical judgement: 17% accuracy (95% CI not reported)	⊕⊙⊙⊙ very low
								Bayer *et al*^30^ Retrospective application of three different screening tools. (Modified) Robson tool: sensitivity 0.95, specificity 0.43, PPV 0.32, NPV 0.97. BAS 90-30-90: sensitivity 0.62, specificity 0.83, PPV 0.51, NPV 0.89. MEWS ≥4 sensitivity 0.74, specificity 0.75, PPV 0.45, NPV 0.91 (95% CI not reported)	
*Sepsis recognition by EMS (use of screening tool not reported)*									
3	963	Non-RCT	Very serious‡	None	Not serious§	Very serious¶	Very serious††	Erwin *et al*^20^ Screening based on SSC criteria. For sepsis: sensitivity 33% (95% CI 18% to 53%), specificity 89% (95% CI 08% to 94%), PPV 50% (95% CI 28% to 72%), NPV 80% (95% CI 70% to 87%). For severe sepsis: sensitivity 20% (95% CI 5% to 51%), specificity 94% (95% CI 87% to 97%), PPV 29% (95% CI 08% to 64%), NPV 91 (95% CI 83% to 95%)	⊕⊙⊙⊙ very low
								Shiuh *et al*^27^ Screening based on SSC criteria, also stratified by lactate, lactate ≤4 ‘sepsis advisory’ while lactate ≥4 ‘sepsis alert’. 74.2% of ‘Sepsis Advisory’ patients and 76.7% of ‘sepsis alert’ patients received a hospital diagnosis of severe infection or sepsis (95% CI not reported)	
								Travers *et al*^28^ Screening criteria not defined. Specificity 78.85% (95% CI 75.23 to 82.17), sensitivity 73.4% (95% CI 61.40 to 83.05), PPV 30.59% (95% CI 23.76 to 38.11), NPV 95.86% (95% CI 93.61 to 97.49), accuracy 78% (52 true positives, 440 true negatives)	

*Seymour *et al* CIS not specific to sepsis (CIS intended to identify all cases of critical illness). Polito *et al* and Bayer *et al* studies limited to single EMS systems, Bayer *et al* physician-based EMS.

†Polito *et al* failed to report CIs, small sample size in Bayer *et al* study.

‡All studies patient selection/eligibility criteria, exposure/outcome reporting, confounding.

§Guerra *et al*, Erwin *et al* and Shiuh *et al* include lactate measurement (not widely available within EMS). In majority of studies, the population limited to single EMS agency/hospital so limited generalisablity. Bayer *et al* used physician-based EMS.

¶All included studies have small sample sizes, thus imprecise point estimates. In several studies, CIs are not reported.

**Guerra *et al* publication bias likely.

††Published in abstract only, unable to reliably critically appraise.

BAS 90-30-90, systolic blood pressure <90 mm Hg; respiratory rate >30 bpm; SpO_2_ <90%; CIS, critical illness score; EMS, Emergency Medical Services; MEWS, Modified Early Warning Score; modified Robson, Robson tool with addition of SpO_2_; non-RCT, non-randomised (observational) study; NPV, negative predictive value; PPV, positive predictive value; PRESEP, Prehospital Early Sepsis Detection; SSC, Surviving Sepsis Campaign.

### Data synthesis

There was considerable variation in the methodological approach adopted across the studies as well as the outcome measures reported. The majority of studies identified involve limited numbers of participants, without control and intervention cohorts. Because of these differences, the studies did not answer a unique research question; thus, meta-analysis was not appropriate. A narrative approach to data synthesis was adopted.

### Derivation of prehospital sepsis screening tools

We identified very low-quality evidence (downgraded for indirectness and imprecision), from three observational studies,^30–32^ addressing derivation of prehospital sepsis screening tools (see table 2). Each of the studies adopted a similar approach to screening tool development. Identification of candidate predictors varied slightly between studies; however, once candidate predictors were identified, all studies used univariate logistic regression to determine which candidate predictors were associated with sepsis, followed by multivariable logistic regression, in a stepwise fashion, to build their respective models. Goodness of fit was assessed by Hosmer-Lemeshow test and model performance determined by calculating the area under the receiver operating characteristic curve.^30–32^ Variables used in each screening tool are shown in table 3. None of the studies included a validation study of their respective screening tools.

**Table 3:** Variables used in screening tools

	Variable												
Author (screening tool)	Respiratory rate*	Heart rate*	Temperature*	LOC†	SpO_2_†	Blood pressure†	Lactate†	Blood glucose†	Skin	CBRT	Dispatch category	Location	Age
Seymour (CIS)	•	•			•	•				•			
Polito (PRESS)			•		•	•					•	•	•
Bayer (PRESEP)	•	•	•		•	•							
Wallgren (Robson tool)	•	•	•	•				•					
Wallgren (BAS 90-30-90)	•				•	•							
McClelland (modified Robson tool)	•	•	•	•	•			•					
Bayer (MEWS)	•	•	•	•		•							
Erwin (unnamed)	•	•	•	•			•		•	•			
Guerra (unnamed)	•	•	•			•	•						
Shiuh (unnamed)	•	•	•				•						

*SIRS criteria.

†Organ dysfunction.

CBRT, capillary bed refill time; CIS, critical illness score; LOC, reduced level of consciousness; MEWS, Modified Early Warning Score; PRESEP, Prehospital Early Sepsis Detection; SIRS, systemic inflammatory response syndrome; SpO_2_, oxygen saturations.

Seymour *et al*^32^ developed the critical illness score to predict the risk critical illness among EMS patients. It was not developed to identify sepsis specifically, although the statistical estimates reported in this review relate to sepsis cases only. Their study used the clinical records of 144 913 EMS patients, of whom 4895 had severe sepsis. Polito *et al*^31^ derived the PRESS score from a population of 66 439 EMS encounters. The sample studied included 555 patients at risk of sepsis, of whom 75 were noted to have severe sepsis, while Bayer *et al*^30^ derived the PRESEP score from a sample of 375 EMS patients, of whom 93 had sepsis (including 60 patients with severe sepsis and 12 patients with septic shock). Accuracy of prehospital sepsis screening tools is presented in table 4.

**Table 4:** Performance of screening tools

Author	Sensitivity	Specificity	PPV	NPV
Seymour (CIS)	0.76 (95% CI 0.75 to 0.77)	Not reported	Not reported	Not reported
Polito (PRESS)	0.85 (95% CI not reported)	0.47 (95% CI not reported)	0.19 (95% CI not reported)	0.96 (95% CI not reported)
Bayer (PRESEP)	0.85 (95% CI 0.77 to 0.92)	0.86 (95% CI 0.82 to 0.90)	0.63 (95% CI not reported)	0.95 (95% CI not reported)
McClelland (sepsis) (modified Robson tool)	0.43 (95% CI 0.28 to 0.58)	0.14 (95% CI 0 to 0.40)	Not reported	Not reported
McClelland (severe sepsis) (modified Robson tool)	0.30 (95% CI 0.12 to 0.47)	0.77 (95% CI 0.60 to 0.95)	Not reported	Not reported
Bayer (modified Robson tool)	0.95 (95% CI not reported)	0.43 (95% CI not reported)	0.32 (95% CI not reported)	0.97 (95% CI not reported)
Wallgren (sepsis) (Robson tool)	0.75 (95% CI not reported)	Not reported	Not reported	Not reported
Wallgren (severe sepsis) (Robson tool)	0.93 (95% CI not reported)	Not reported	Not reported	Not reported
Bayer (BAS 90-30-90)	0.62 (95% CI not reported)	0.83 (95% CI not reported)	0.51 (95% CI not reported)	0.89 (95% CI not reported)
Wallgren (sepsis) (BAS 90-30-90)	0.73 (95% CI not reported)	Not reported	Not reported	Not reported
Wallgren (severe sepsis) (BAS 90-30-90)	0.70 (95% CI not reported)	Not reported	Not reported	Not reported
Bayer (MEWS)	0.74 (95% CI not reported)	0.75 (95% CI not reported)	0.45 (95% CI not reported)	0.91 (95% CI not reported)
Guerra	0.48 (95% CI not reported)	Not reported	Not reported	Not reported
Erwin (sepsis)	0.33 (95% CI 0.18 to 0.53)	0.89 (95% CI 0.08 to 0.94)	0.50 (95% CI 0.28 to 0.72)	0.80 (95% CI 0.70 to 0.87)
Erwin (severe sepsis)	0.20 (95% CI 0.05 to 0.51)	0.94 (95% CI 0.87 to 0.97)	0.29 (95% CI 0.08 to 0.64)	0.91 (95% CI 0.83 to 0.95)
Shiuh	0.75 (95% CI not reported)	Not reported	Not reported	Not reported
Travers	0.73 (95% CI 0.61 to 0.83)	0.79 (95% CI 0.75 to 0.82)	0.31 (95% CI 0.24 to 0.38)	0.96 (95% CI 0.94 to 0.98)

CIS, critical illness score; MEWS, Modified Early Warning Score; PRESEP, Prehospital Early Sepsis Detection.

### Sepsis recognition by EMS (using a screening tool)

We identified very low-quality evidence (downgraded for risk of bias, indirectness and imprecision), from two observational studies,^12^ ^15^ addressing recognition of sepsis by EMS personnel using a screening tool (see table 2). Guerra *et al*^12^ report that emergency medical technicians (EMTs) trained to recognise sepsis correctly identified 32/67 (48%) patients with sepsis, with failure to recognise sepsis in 35/67 (52%) of cases; however, this figure may be misleading. In 5/35 (14%) of cases, the patient's vital signs did not meet the criteria of the sepsis screening tool while in EMS care; in 8/35 (23%) of cases, the patients had cryptic shock but EMTs did not have lactate meters; and in 13/35 (37%) of cases, diagnosis was made by abnormal white cell count (only available in hospital). In 9/35 (26%) of cases, EMTs failed to identify sepsis when sufficient diagnostic criteria were available to them. The high proportion of patients missed due to lack of white cell count highlights a limitation of prehospital sepsis screening tools. Guerra *et al*^12^ further reported that among patients with sepsis transported by EMS crews not trained to recognise sepsis, 5/45 (11%) were identified as patients with sepsis.

McClelland and Jones^15^ scrutinised the records of all patients with sepsis conveyed by a regional ambulance service to a university hospital to determine whether ambulance clinicians, previously trained in the use of a screening tool, recognised and documented suspected sepsis. The screening tool used was based on the Robson tool amended to include oxygen saturations as an indicator of organ dysfunction. The authors concluded that the use of the screening tool by ambulance clinicians was inconsistent but improved sepsis recognition.

### Retrospective application of EMS data to screening tool by researcher

We identified very low-quality evidence (downgraded for risk of bias, indirectness and imprecision), from two observational studies,^21^ ^30^ addressing retrospective application of prehospital data to screening tools (see table 2). Wallgren *et al*^21^ compared two screening tools (Robson tool and BAS 90-30-90 score) with EMS clinician judgement. The Robson tool performed better than the BAS 90-30-90 score (see table 4). Clinician judgement, defined as ‘documentation of suspected sepsis, septicaemia, urosepsis or blood poisoning in the patient's clinical record’, was reported to be 11.9% and 16.9% sensitive for sepsis and severe sepsis, respectively. CIs were not reported.

Bayer *et al*^30^ compared the performance of their PRESEP score with the MEWS, BAS 90-30-90 and Robson tool reporting that the PRESEP score surpassed MEWS and BAS 90-30-90 for sensitivity, speciﬁcity, positive predictive value (PPV) and negative predictive value (NPV). The Robson tool showed better sensitivity; however, the PRESEP tool had better specificity. Furthermore, the PRESEP score showed better PPV and comparable NPV than the Robson tool (see table 4).

### Sepsis recognition by EMS (use of screening tool not reported)

We identified very low-quality evidence (downgraded for risk of bias, indirectness, imprecision and abstract only publication), from three observational studies,^20^ ^27^ ^28^ addressing accuracy of paramedic diagnosis of sepsis in clinical practice (see table 2). All three studies were published in abstract and it is not clear if paramedics used a screening tool or if they received any training to improve sepsis recognition.

Erwin *et al*^20^ compared paramedic diagnosis of sepsis and severe sepsis with physician diagnosis (see table 4). The level of agreement between paramedics and physicians was low (κ=0.25 and 0.16, respectively). These results lead the authors to conclude that sepsis criteria were more useful for ruling-out sepsis than diagnosing sepsis.

In the study by Shiuh *et al*,^27^ EMS crews stratified patients with sepsis according to prehospital lactate readings. If patients had a lactate >4 mmol/L, paramedic crews provided the hospital with an ‘alert’ message, whereas if the lactate was in the range of 2.5–3.9 mmol/L, they provided the hospital with an ‘inform’ message prior to, or on, hospital arrival. They reported data for 219 patients with sepsis for whom a lactate reading was available; they did not report data for those patients where a lactate reading was not known/unavailable (see table 4).

Travers *et al*^28^ compared accuracy of paramedic sepsis diagnosis in 629 cases. Thermometry was not available to paramedics to confirm body temperature with any degree of accuracy. Paramedic diagnosis agreed with physician diagnosis in 78% of cases. This is the largest paramedic diagnostic accuracy study, but unfortunately detail is lacking.

## Discussion

The studies identified provide low-quality or very low-quality evidence to suggest that accuracy of prehospital sepsis recognition by ambulance clinicians varies considerably. This variation could have numerous causes. In many areas, paramedic education programmes have not focused sufficient attention on sepsis as a clinical syndrome and paramedic knowledge of sepsis is often poor.^5^ ^39–41^ It is possible that ambulance clinicians encounter patients with sepsis earlier in their clinical course, before they become seriously ill, and it is also not known if in-hospital and prehospital clinical assessments, such as blood pressure, correlate in patients with sepsis. An additional factor may be that routine in-hospital tests such as white cell count and lactate are not commonly used within EMS, which may limit the ability to extrapolate from in-hospital studies.

The majority of the prehospital sepsis screening tools rely upon the Surviving Sepsis Campaign systemic inflammatory response syndrome (SIRS) criteria which were initially described to improve sepsis recognition in the ED and intensive care environments. Although SIRS describe physiological signs marking the transition from infection to sepsis, they lack specificity for sepsis. SIRS are observable following a wide variety of insults other than infection, leading some to question the value of SIRS to identify sepsis.^42^ ^43^ Churpek *et al*^44^ recently demonstrated that SIRS criteria were not reliable predictors of sepsis or mortality in the ward setting. Use of SIRS criteria to identify sepsis in the prehospital environment may therefore be equally ineffective.

The three studies documenting the development of prehospital screening tools for sepsis included more organ dysfunction criteria and also included non-SIRS variables (see table 3). Among these, tools sensitivity for severe sepsis ranged from 0.76 to 0.85, while specificity ranged from 0.47 to 0.86; they appear to perform better than tools based on the SIRS diagnostic criteria (see table 4); however, none have been clinically validated.

Although nine studies were identified in the course of this review, only five were concerned with screening of patients in clinical practice by EMS clinicians.^12^ ^15^ ^20^ ^27^ ^28^ These studies enrolled a total of 1123 patients, over half of whom (675) were in the Travers *et al*^28^ study. Given the very limited number of participants in the remaining studies (range 49–183), it is unlikely that reported point estimates are sufficiently precise to draw conclusions with confidence.

## Conclusion

The identified studies indicate that sepsis recognition within EMS is highly variable. The majority of screening tools studied in clinical practice favour SIRS criteria which may limit the specificity of these tools. Screening tools derived from EMS data have been developed; these tools appear to include more organ dysfunction variables. Retrospective application of ambulance data to these EMS-derived tools suggests that they may help improve sepsis recognition as they demonstrate similar sensitivity with greater specificity. There is a need to undertake validation studies of EMS-derived sepsis screening tools to determine their efficacy. It remains to be seen if use of a prehospital sepsis screening tool provides any significant clinical benefit.
