# Supplementary material for: Identification of adults with sepsis in the prehospital environment: a systematic review
Source: BMJ Open. 2016 Aug 5;6(8):e011218. doi: 10.1136/bmjopen-2016-011218 (PMC4985978; doi:10.1136/bmjopen-2016-011218)
Supplement: supplementary appendix [file bmjopen-2016-011218supp_appendix.pdf]

Appendix 1

MEDLINE search strategy

1 exp Sepsis/di

2 septic.mp.

3 septic*.mp.

4 septic?emia.mp.

5 SIRS.mp.

6 sepsis.m_titl.

7 (septic adj shock).mp.

8 exp Systemic Inflammatory Response Syndrome/ or SIRS.mp.

9 (systemic adj inflammatory adj response adj syndrome).mp

10 (hypotension adj induced adj hypoperfusion).mp

11 (hypotension adj induced adj hypoperfusion).m_titl.

12 (cryptic adj shock).mp.

13 septic shock.mp. or Shock, Septic/

14 (bacterial adj infection).mp.

15 1 or 2 or 3 or 4 or 5 or 6 or 7 or 8 or 9 or 10 or 11 or 12 or 13 or 14

16 (emergency adj medical adj service*).mp.

17 (emergency adj medical adj service*).m_titl.

18 exp Emergency Medical Services/ or EMS.mp.

19 HEMS.mp.

20 HEMS.m_titl.

21 (emergency adj medical adj technician*).mp.

22 (emergency adj medical adj technician*).m_titl.

23 exp Emergency Medical Technicians/ or emt*.mp.

24 emt.m_titl.

25 paramedic*.mp.

26 "paramedic*".m_titl.

27 (pre-hospital or prehospital).mp.

28 (pre-hospital or prehospital).m_titl.

29 (pre adj hospital).mp

30 pre?hospital.mp.

31 out-of-hospital.mp.

32 (out adj of adj hospital).mp.

33 OOH.mp.

34 exp Air Ambulances/ or ambulance*.mp. or exp Ambulances/

35 16 or 17 or 18 or 19 or 20 or 21 or 22 or 23 or 24 or 25 or 26 or 27 or 28 or 29 or 30 or 31 or 32 or 33 or 34

36 15 and 35
