# Supplementary material for: Identification of adults with sepsis in the prehospital environment: a systematic review
Source: BMJ Open. 2016 Aug 5;6(8):e011218. doi: 10.1136/bmjopen-2016-011218 (PMC4985978; doi:10.1136/bmjopen-2016-011218)
Supplement: supplementary material [file bmjopen-2016-011218supp_material.pdf]

Prehospital sepsis screening: a systematic review (Supplementary material).

<table>
<tr><th>Author (year), country</th><th>Study design (n)</th><th>Manuscript type</th><th>Study Population</th><th>Study Intervention</th><th>Key findings</th><th>Quality Assessment/Limitations</th></tr>
<tr><td>Seymour et al. (2010), USA</td><td>Non-RCT n=144 913</td><td>Paper</td><td>Non-trauma, non-cardiac arrest adult patients transported to hospital by EMS</td><td>Development of a screening tool (Critical Illness Score) utilising retrospective EMS/hospital data</td><td>Weighted score calculated using age, systolic blood pressure, respiratory rate, GCS, pulse oximetry, nursing home as place of residence. Sensitivity (severe sepsis) 0.76 (95% CI, 0.75-0.77).</td><td>Risk of bias Low
Inconsistency None
Indirectness Critical Illness Score not specific to sepsis
Imprecision None
Other None</td></tr>
<tr><td>Polito et al. (2015), USA</td><td>Non-RCT n=555</td><td>Paper</td><td>Non-trauma, non-cardiac arrest, non-pregnant, non-psychiatric, non-toxic OD adult patients transported to hospital by EMS</td><td>Development of a sepsis screening tool (PRESS score) utilising retrospective EMS/hospital data</td><td>Weighted score calculated using ‘sick person’ dispatch category, tactile temperature, systolic blood pressure, pulse oximetry. Sensitivity 85%, specificity 47%, PPV 19%, NPV 96%. Confidence intervals not reported.</td><td>Risk of bias Low
Inconsistency None
Indirectness Study limited to single EMS and hospital system.
Imprecision Failed to report confidence intervals in all areas
Other None</td></tr>
<tr><td>Bayer et al. (2015), Germany</td><td>Non-RCT n=375</td><td>Paper</td><td>Consecutive adult EMS patients admitted to a single hospital. Prehospital physician based EMS system</td><td>Development of a sepsis screening tool (PRESEP score) utilising retrospective EMS/hospital data</td><td>Weighted score calculated using heart rate, respiratory rate, pulse oximetry, systolic blood pressure. Sensitivity 0.85 (95% CI 0.77 to 0.92), Specificity 0.86 (95% CI 0.82 to 0.90), PPV 0.66, NPV 0.95. Also ‘bench tested’ other tools:
<table><tr><td>Measure</td><td>MEWS≥4</td><td>BAS 90-30-90</td><td>modified Robson tool</td></tr><tr><td>Sensitivity</td><td>0.74</td><td>0.62</td><td>0.95</td></tr><tr><td>Specificity</td><td>0.75</td><td>0.83</td><td>0.43</td></tr><tr><td>PPV</td><td>0.45</td><td>0.51</td><td>0.32</td></tr><tr><td>NPV</td><td>0.91</td><td>0.89</td><td>0.97</td></tr></table></td><td>Risk of bias Low
Inconsistency None
Indirectness Study limited to single physician EMS and hospital system.
Imprecision Small sample size limits precision
Other None</td></tr>
</table>

Prehospital sepsis screening: a systematic review (Supplementary material).

Erwin *et al.* (2011), USA	Non-RCT n=151	Abstract	Patients transported by EMS from 3 nursing homes	EMS patients screened for no-sepsis, sepsis, severe sepsis using screening tool provided and point of care lactate	| | *Sepsis* | *Severe sepsis* | *Sensitivity*: 0.33 (95%CI 0.18-0.53); 0.20 (95%CI 0.05-0.51)<br>*Specificity*: 0.89 (95%CI 0.80-0.94); 0.94 (95%CI 0.87-0.97)<br>*PPV*: 0.50 (95%CI 0.28-0.72); 0.29 (95%CI 0.08-0.64)<br>*NPV*: 0.80 (95%CI 0.70-0.87); 0.91 (95%CI 0.83-0.95)	*Risk of bias* High<br>*Inconsistency* None<br>*Indirectness* Study limited to single EMS and hospital system. Very specific patient population. Lactate not widely available within EMS.<br>*Imprecision* Small sample size limits precision<br>*Other* Abstract publication
Shiuh *et al.* (2012), USA	Non-RCT n=183	Abstract	Out of hospital patients who had venous lactate measured	Utility of prehospital lactate in sepsis	183 EMS patients with lactate readings<br>*"Sepsis Advisory" (lactate 2.5-3.9mmol/l)* 66/86 (76.7%)<br>*"Sepsis Alert" (lactate >4mmol/L)* 72/97 (74.2%)	*Risk of bias* High<br>*Inconsistency* None<br>*Indirectness* Study limited to single EMS and hospital system. Lactate not widely available within EMS<br>*Imprecision* Small sample size limits precision. Failed to report appropriate statistical tests<br>*Other* Abstract publication
Guerra *et al.* (2013), USA	Non-RCT n=112	Paper	Severe sepsis patients admitted to hospital following transport by EMS to any of to 3 participating ED's.	Paramedic accuracy of sepsis diagnosis following the introduction of sepsis recognition training	*Identified by trained providers* 32/67<br>*Missed by trained providers* 35/67: *Normal vital signs* 5/35; *Cryptic shock* 8/35; *Elevated WCC* 13/35; *"true failure"* 9/35<br>*Identified by untrained providers* 5/45<br>*Missed by untrained providers* 40/45	*Risk of bias* High<br>*Inconsistency* None<br>*Indirectness* Lactate not widely available within EMS<br>*Imprecision* Small sample size limits precision. Failed to report confidence intervals.<br>*Other* Publication bias
Travers *et al.* (2013), Canada	Non-RCT n=629	Abstract	EMS patients screened by paramedics for sepsis	Comparison of paramedic and physician diagnosis	*Sensitivity* 73.24 (95% CI 61.40-83.05%)<br>*Specificity* 78.85% (95% CI 75.23-82.17%)<br>*Accuracy* 78% (429/629; 52 true positive, 440 true negative)<br>*PPV* 30.59% (95% CI 23.76-38.11%)<br>*NPV* 95.86% (95% CI 93.61-97.49%)<br>*PLR* 3.46 (95% CI 2.80-4.29)<br>*NLR* 0.34 (95% 0.23-0.50)	*Risk of bias* Unclear<br>*Inconsistency* None<br>*Indirectness* Study limited to single EMS and hospital system.<br>*Imprecision* None<br>*Other* Abstract publication

Wallgren *et al.* (2014), Sweden	Non-RCT n=353	Paper	Sepsis patients (identified by hospital ICD code) transported by EMS	Comparison of Robson tool, BAS 90-30-90 and EMT documentation of clinical impression	'bench test' of clinical tools by researcher<br>*sepsis*<br>*EMT records* / *BAS 90-30-90* / *Robson tool*<br>*Sensitivity* 11.9% / 43.4% / 75%<br>*Severe sepsis*<br>*EMT records* / *BAS 90-30-90* / *Robson tool*<br>*Sensitivity* 16.9% / 70.4% / 92.9%	*Risk of bias* High<br>*Inconsistency* None<br>*Indirectness* Retrospective application of tools by researcher. Study data from single EMS and hospital system.<br>*Imprecision* Small sample size limits precision. Failed to report confidence intervals.<br>*Other* None
McClelland and Jones (2015), UK	Non-RCT n=49	Paper	Sepsis patients (identified by hospital ICD code) transported by single EMS agency to single hospital	Proportion of sepsis patients identified by EMS crews utilising a modified Robson Screening tool	*Sepsis*<br>18/42 patients. Sensitivity 43% (95%CI 28-58%), Specificity 14% (95%CI 0-40%)<br>*Severe sepsis*<br>8/27 patients. Sensitivity 30% (95%CI 12-47%), Specificity 77% (95%CI 60-95%).	*Risk of bias* High<br>*Inconsistency* None<br>*Indirectness* Study limited to single EMS and hospital system.<br>*Imprecision* Small sample size limits precision<br>*Other* None

non-RCT: non-randomised controlled trial (observational study), EMS: Emergency Medical Services, OD: overdose, ED: emergency department, ICD: International Classification of Disease, EMT: Emergency Medical Technician, GCS: Glasgow Coma Score, 95%CI: 95% confidence interval, PPV: positive predictive value, NPV: negative predictive value, PLR: positive likelihood ratio, NLR: negative likelihood ratio, WCC: white cell count, BAS 90-30-90: Swedish score to identify sepsis, MEWS: modified early warning score.

Characteristics of included studies

Prehospital sepsis screening: a systematic review (Supplementary material).
